# The Protective Effects of Beta-Casomorphin-7 against Glucose -Induced Renal Oxidative Stress In Vivo and Vitro

**DOI:** 10.1371/journal.pone.0063472

**Published:** 2013-05-03

**Authors:** Wei Zhang, Jinfeng Miao, Shanshan Wang, Yuanshu Zhang

**Affiliations:** Key Lab of Animal Physiology and Biochemistry, Ministry of Agriculture, Nanjing Agriculture University, Nanjing, People's Republic of China; Case Western Reserve University, United States of America

## Abstract

Oxidative stress is implicated in the pathogenesis of diabetic nephropathy. The present study aimed to investigate the effect of β-casomorphin-7 (BCM7) on the oxidative stress occurring in kidney tissue in streptozotocin (STZ)-induced diabetic rats and proximal tubular epithelial cells (NRK-52E) exposure to high glucose (HG) by using biochemical methods. There is a significant decrease in plasma insulin and a significant increase in plasma glucagon in the rats of diabetic group. Oral administration of BCM7 for 30 days to rats with STZ-induced diabetes resulted in a significant increase in serum level of insulin, and a decrease in the level of glucagon. Moreover, rats with STZ-induced diabetes had lower levels of superoxide dismutase (SOD), glutathione peroxidase (GPx) and total antioxidative capacity (T-AOC), higher levels of malondialdehyde (MDA) and hydrogen peroxide (H_2_O_2_) in the kidney than that in the control rats. The administration of BCM7 altered the changes of SOD, GPx, T-AOC, MDA and H_2_O_2_ in the kidney of diabetic rats. Furthermore, BCM7 alleviated high glucose-induced decreasement in SOD and GPx activity, increasement in MDA contents in the NRK-52E cells. BCM7 ameliorated the changes of angiotensin converting enzyme (ACE) and ACE2 levels in the kidney of diabetic rats and BCM7 lowered the levels of angiotensin (Ang)II in the kidney of diabetic rats and culture medium for cells. Moreover losartan (antagonist of angiotensin II type I receptor) lowered the high glucose-induced oxidative stress in the NRK-52E cells. Our results suggest that administration of BCM7 would alleviate high glucose-induced renal oxidative stress in vivo and in vitro, which may be associated with down regulation of the concentration of Ang II partly.

## Introduction

Diabetes mellitus (DM) is a health problem affecting millions of individuals worldwide[1]. Diabetic nephropathy (DN), as an important complication of DM, has become the most common cause of end-stage renal failure among patients [2], [3].

It has been suggested that oxidative stress constitute the key and common events in the pathogenesis of DN[4]. Brownlee had raised up that oxidative stress resulting from increased production of ROS is a unifying mechanism of diabetic complications, including diabetic nephropathy[5]. Oxidative stress has been implicated early and late events of DN. While antioxidant administration has been shown to have potential beneficial effects in the human kidney and experimental diabetes [6]–[8]. To prevent the development and progression of DN, an appropriate way to alleviate or inhibit the oxidative stress of kidney may be helpful.

β-casomorphins-7, opioid-like peptides formed during proteolytic degradation of human and animal β-casein[9], Bovine β-casomorphin-7 (Tyr-Pro-Phe-Pro-Gly-Pro-Ile, BCM7) are fragments f60-66 of β-casein[10]. Our previous experiments have demonstrated that BCM7 had a hypoglycemic effect in normal and diabetic rats and reduced the absorption of glucose in the small intestine of rats [11], [12].BCM7 also can reduce the oxidative stress of diabetic rats[12].Moreover BCM7 can attenuate kidney damage and renal interstitial fibrosis caused by diabetes[13]. However, there are no publications testing the effects of BCM7 on diabetic renal oxidative stress. In the present study, we investigate whether management with BCM7 has any protective effects on diabetic renal oxidative stress in STZ-induced diabetic rats. We also investigate the potential mechanism by which BCM7 exerts its effects.

## Materials and Methods

### Drugs and peptides

Streptozotocin (STZ) was purchased from Sigma-Aldrich China (Shanghai, China). Bovine BCM7 (Tyr-Pro-Phe-Pro-Gly-Pro-Ile) was obtained from GL Biochem (Shanghai, China). The kits for superoxide dismutase (SOD), glutathione peroxidase (GPx), total antioxidant capacity (T-AOC), hydrogen peroxide (H_2_O_2_) and malondialdehyde (MDA) were purchased from Nanjing Jiancheng Biochemistry Reagent Co (NJBC, Nanjing, China). Radioimmunoassay kits for insulin, glucagon and AngII was purchased from Beijing Beifang Pharmaceutical Co. (Beijing, China). All of the other chemicals and reagents were standard commercially available biochemical quality.

### Animals

Male Sprague-Dawley (SD) rats weighing 200 g±10 g were purchased from Shanghai Laboratory Animal Center, Chinese Academy of Science (SLAC, CAS) and housed in a controlled environment with a 12 h light-dark cycle. All animal care and procedures were in accordance with national and institutional policies for animal health and well-being. All rat samples collection and field study were approved by guide for care and use of laboratory animals of Nanjing Agriculture University (Nanjing, China) and the Jiangsu Provincial Academy of Agricultural Sciences. The license number was SCXK (Su) 2002-0029. All efforts were made to minimize the number of animals used and their suffering. The animals were acclimatized for 1 week before the study and had free access to water and standard rat chow throughout the experiment.

### Experimental protocol

The rats were divided into three groups comprising of eight animals in each group as follows:

Group C: normal rats, oral given distilled water (7.5×10^−6^ mol/day/kg of body weight) by gavage for 30 days.

Group D: diabetic rats, oral given distilled water (7.5×10^−6^ mol/day/kg of body weight) by gavage for 30 days.

Group BCM7: diabetic rats, oral given BCM7 (7.5×10^−6^ mol/day/kg of body weight) by gavage for 30 days.

Diabetes was induced by intraperitoneal injection of STZ dissolved in 0.1 mol/L sodium citrate buffer (pH = 4.0) at a dose of 60 mg/kg body weight. Fasting, tail-vein, blood glucose was measured three days after injection by using one touch glucomter (YiCheng, BeiJing, China), and rats that had a glucose level over 16.7 mmol/L were considered to be diabetic rats [14].To investigate the preventive effects of BCM7 treatment, BCM7 treatment were carried out 2 weeks after the injection of STZ. All rats were euthanized after 30 days of treatment.

On day 30, the animals were fasted overnight. Blood glucose, body and kidney weights were measured. Serum, plasma and kidney were collected for future analyses. All samples were stored at −70°C.

### Cell culture

The normal rat kidney proximal tubular epithelial cell line NRK-52E was purchased from the American Type Culture Collection (ATCC, Rockville, MD,USA). Cells were grown in Dulbecco's modified Eagles medium (DMEM, Wisent) containing 5.5 mmol/L D-glucose (normal glucose, NG) supplemented with 10% fetal bovine serum (FBS) (containing100 IU/mL penicillin and 100 µg/mL streptomycin) at 37°C in a humidified atmosphere of 5% CO2. Cells were maintained in serum-free media for 24 h before each experiment.

The high glucose (HG) medium contained 30 mmol/L D-glucose, whereas normal glucose DMEM media supplemented with 24.5 mM mannitol was served as osmotic control (HM) in the experiment. β-casomorphin-7 (BCM7,10^−5^/10^−7^/10^−9^ mol/L) was added to the cell cultures 30 min prior to changing the medium to a high glucose medium. To assess the effect of losartan (antagonist of angiotensinII type I receptor) on high glucose-induced oxidative stress of NRK-52E cells, losartan were added to cell cultures 15 min prior to changing the medium to a high glucose medium. Each treatment was repeated in triplet for the following analysis.

### Measurements of serum insulin and glucagon

The concentrations of serum insulin and glucagon were determined using the commercial radioimmunoassay (RIA) kit and the intra- and inter-assay coefficients of variation were less than 10% and 15% respectively.

### Preparation of renal homogenate

Renal samples (100 mg) were chipped and homogenized in a nine-time volume of ice-cold isotonic saline, and then the homogenates were centrifuged at 12000×g for 10 min at 4°C to remove any cell debris. Protein concentration of the supernatant was estimated using the method of coomassie light blue [15].

### Evaluation of renal antioxidant assays

#### SOD assay

SOD activity in the kidney and NRK-52E cells was measured according to the method of [16], [17] using a kit (NJBC, Nanjing, China). This kit utilizes a tetrazolium salt for the detection of superoxide radicals generated by xanthine oxidase and hypoxanthine to form a red formazan dye, which was measured at the optical density at 550 nm using a spectrophotometer. The enzyme activity was expressed as U/mg protein and 1 unit of enzyme is defined as the enzyme activity that inhibits autoxidation of pyrogallol by 50%.

#### GPx assay

GPx activity in the kidney and NRK-52E cells was assayed by a method previously described [18], [19] through a coupled assay using H_2_O_2_ and dithio-bis-nitrobenzoic acid (DTNB).One unit of enzyme activity of the enzymes unit represents a decrease in GSH concentration of 1 µmol/L/min after subtraction of non-enzymic mode. All measurements were performed in triplicates and results were normalized per 1 mg protein.

#### T-AOC

The renal total antioxidative capacity (T-AOC) was measured with a commercial kit (NJBC, Nanjing, China). This assay measures the ferric reducing ability of the supernatant. The stable color of the Fe^2+^-o-phenanthroline complex (produced by the reducing agents in kidney reducing Fe^3+^ to Fe^2+^, which reacts with the substrate o-phenanthroline) was measured at 520 nm. T-AOC was expressed in U/mg protein where 1 U is defined as an increase in absorbance (A520) of 0.01/min/mg protein at 37°C.

### Measurements of renal oxidative stress

#### MDA level

The MDA contents in the kidney and NRK-52E cells were measured by the thiobarbituric acid method [20]–[22], with commercially available kits following the manufacturer's instruction (NJBC, Nanjing, China). The samples were detected by dual wavelength using 450 nm wavelength in order to eliminate the influence of glycation and part of other lipidic aldehydes. All measurements were performed in triplicate, and the results were expressed as nmol of MDA per mg protein.

#### H_2_O_2_ level

Hydrogen peroxide in cytosolic extracts was measured using a commercial kit (NJBC, Nanjing, China) following the manufacturer's instructions as previously described [23].

### Radioimmunoassay for Ang II

The concentration of Ang II in renal homogenate and culture medium (10%, contain10 ul 0.3 M Na_2_EDTA, 5 ul 0.32 M dimercaptopropanol and 10 ul 0.34 M 8-hydroxyquinoline/ml homogenate) was measured by radioimmunoassay. The kits were purchased from Beijing North Institute of Biological Technology and the intra- and inter-assay coefficients of variation were less than 10%and 15%.

### Real-time polymerase chain reaction (qPCR) analysis

Total RNA was extracted from kidney of rat using Trizol reagent according to the manufacturer's instructions (Takara, Dalian, China). Total RNA (2 µg) was subjected to first-strand cDNA synthesis using random primer, M-MLV reverse transcriptase and RNase inhibitor (SunShine Bio, nanjing, China). The specific primers for the polymerase chain reaction (PCR) were as follows: The specific primer sequences for ACE, ACE2 and β-actin were designed with the software program Primer Express (PE, Applied Biosystems Inc., Foster City, USA) and given in Table 1. The SYBR Green quantitative real-time PCR Master Mix (Roche, Mannheim, Germany) was used to quantify the relative abundance of target mRNA. The qPCR was performed according to manufacturer's instructions and the accumulated fluorescence was detected using a real-time PCR detection system (Prism 7300; Applied Bio systems Inc., Foster City, USA).β-actin was served as the endogenous control. In order to calculate differences in the expression level of each target gene, the ΔΔCT method for relative quantification was used according to the manufacturer's manual.

**Table 1:** Primers sequences for ACE, ACE2 and β-actin.

gene	Genbank accession number	Primers sequence(5′-3′)	Orientation	Product size
ACE	NM012544	ATGCCTCTGCGTGGGACTTC	Forward	112 bp
		TACTGCACGTGGCCCATCTC	Reverse	
ACE2	AY881244	AATCGTAGGCTCTGGGCTTGG	Forward	182 bp
		TTCGATCAACTGGTTTCGGTTGTA	Reverse	
β-actin	AF122902	CCCTGTGCTGCTCACCGA	Forward	198 bp
		ACAGTGTGGGTGACCCCGTC	Reverse	

### Western-blot analysis

The proteins of kidney for Western-blot were extracted with a protein extraction kit that was purchased from Beyotime Institute of Biotechnology (Haimen, China) Protein (80 µg) was run by SDS-PAGE on a 8% gel, and transblotted onto polyvinylidene difluoride membranes (PVDF, Millipore, Bedford, MA, USA). At the end of the transfer, the membranes were blocked with 5% nonfat skim milk powder in tris-buffered saline and 0.1% Tween-20 (TBST) for 1 hour at room temperature and then incubated overnight at 4°C with primary antibodies of ACE2/ACE (1∶300; Santa Cruz Biotechnology, CA, USA°C)/β-actin antibody (1∶10000; Cell Signaling, Beverly, USA). Immunoreactive bands were detected with horseradish peroxidase-conjugated secondary antibodies (1∶10000, abcam, MA, USA) and an enhanced chemiluminescence system (Pierce, Rockford, LA, USA). The density of individual bands was quantified by densitometric scanning of the blots using Quantity One software (Bio-Rad, Hercules, CA, USA).

### Statistical analysis

Data are expressed as the mean±SEM. Data were compared by one-way ANOVA with least significant difference (LSD) post-hoc tests used to compare individual means as appropriate. P<0.05 was considered significant. All statistical analyses were performed using SPSS16.0 software (SPSS, Chicago, IL, USA).

## Results

### General measurements

**Figure 1:**
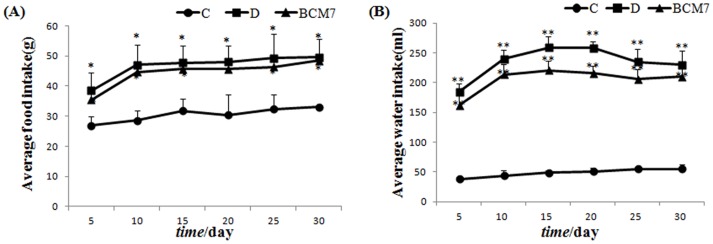
Effects of BCM7 on average food intake (A) and water intake (B) in rats (n = 8). Data are expressed as mean±SEM. (C) Control group; (D) Diabetic group; (BCM7) β-casomorphin-7 treatment group. *P<0.05, **P<0.01 comparison between C group and D group.

**Figure 2:**
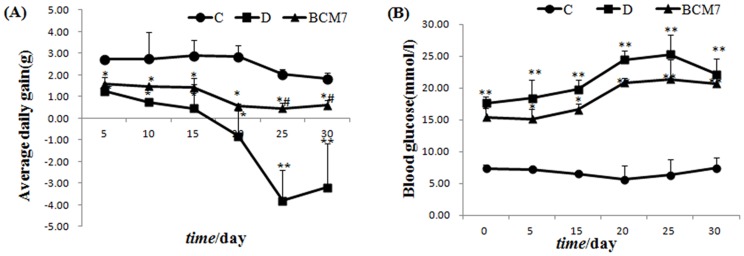
Effects of BCM7 on average daily gain (A) and plasma glucose (B) in rats (n = 8). Data are expressed as mean±SEM (n = 8). (C) Control group; (D) Diabetic group; (BCM7) β-casomorphin-7 treatment group. *P<0.05, **P<0.01 comparison between C group and D group. # P<0.01, ## P<0.01 comparison between D group and BCM7.

As shown in Fig. 1 and Fig. 2, compared with the control group (Group C), the food intake, water intake and blood glucose level increased significantly in the diabetic group (Group D), while the average daily gain decreased significantly. Oral administration of BCM7 in diabetic rats did not modify any of these parameters except the average daily gain significantly.

### Changes of plasma insulin and glucagon

**Figure 3:**
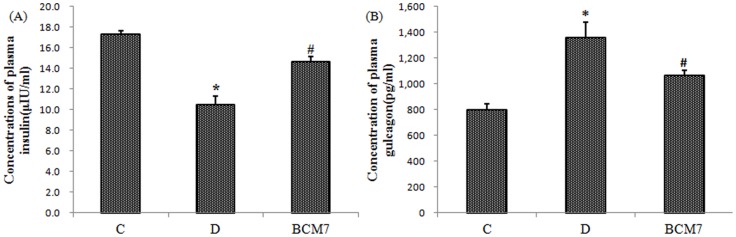
Effects of BCM7 on plasma insulin (A) and plasma glucagon (B) in rats (n = 8). Data are expressed as mean±SEM (n = 8). (C) Control group; (D) Diabetic group; (BCM7) β-casomorphin-7 treatment group. *P<0.05, comparison between C group and D group. #P<0.05, comparison between D group and BCM7.

As shown in Fig. 3, compared with the normal rats, there is a significant decrease in plasma insulin and a significant increase in plasma glucagon in the rats of diabetic group (p<0.05). BCM7 treatment increased plasma insulin and decreased plasma glucagon of diabetic rats (p<0.05).

### Effect of BCM7 on STZ -induced changes in SOD, GPx and T-AOC

Table 2 shows the SOD, GPx and T-AOC in animals treated with STZ, which were decreased in kidney as compared to the control rats (group C). SOD, GPx and T-AOC in the kidney were increased in BCM7 treated diabetic rats (group BCM7) when compared with diabetic control rats (group D).

**Table 2:** Effect of BCM7 on SOD, GPx, T-AOC, MDA and H_2_O_2_ in kidney of rats.

Groups	SOD	GPx	T-AOC	MDA	H_2_O_2_
C	98.27±25.21	85.6±3.17	1.812±0.369	1.00±0.13	17.47±9.53
D	51.78±12.51**	67.7±3.78**	1.48±0.199	1.47±0.04**	21.92±1.19
BCM7	86.45±21.04##	78.4±1.96#	2.031±0.183	1.30±0.14#	20.41±1.09

SOD(U/mg protein),GPx(units/mg protein),T-AOC(U/mg protein),MDA(nmol/mg protein), H_2_O_2_(U/mg prote.in).

(C) Control group; (D) Diabetic group; (BCM7) β-casomorphin-7 treatment group.

Comparison between D group and C group: **P<0.01; Comparison between BCM7 group and D group: #P<0.05, ##P<0.01.

### Effect of BCM7 on STZ-induced changes in MDA and H_2_O_2_ contents

The levels of MDA and H_2_O_2_ in the renal tissue were increased in diabetic group compared with these in the control group. BCM7 treatment reduced MDA (significantly) and H_2_O_2_ (non-significantly) level in the kidney compared to the diabetic rats. (Table 2)

### BCM7 reduced HG-induced oxidative stress in NRK-52E

**Figure 4:**
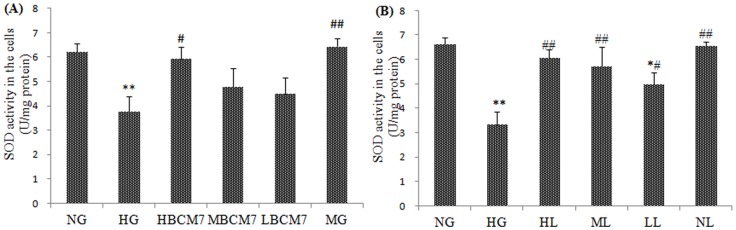
SOD activity in the NRK-52E cells (n = 4). NG(normal glucose), HG(high glucose), HM(5.5 mmol/L glucose plus 24.5 mmol/L mannitol), HBCM7(high glucose plus 10^−5^ mol/L β-casomorphin-7), MBCM7(high glucose plus 10^−7^ mol/L β-casomorphin-7), LBCM7(high glucose plus 10^−9^ mol/L β-casomorphin-7), HL(high glucose plus 10^−4^ mol/L losartan), ML(high glucose plus 10^−5^ mol/L losartan), LL(high glucose plus 10^−6^ mol/L losartan), NL(normal glucose plus 10^−4^ mol/L losartan). Data are mean ± SEM (n = 4 for each group). *P<0.05, **P<0.01 compared with NG, #P<0.05, ##P<0.01 compared with HG.

**Figure 5:**
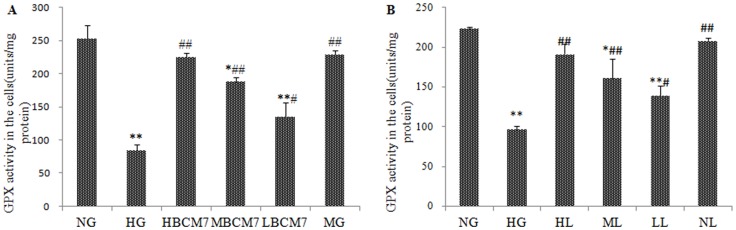
GPx activity in the NRK-52E cells (n = 4). NG(normal glucose), HG(high glucose), HM(5.5 mmol/L glucose plus 24.5 mmol/L mannitol), HBCM7(high glucose plus 10^−5^ mol/L β-casomorphin-7), MBCM7(high glucose plus 10^−7^ mol/L β-casomorphin-7), LBCM7(high glucose plus 10^−9^ mol/L β-casomorphin-7), HL(high glucose plus 10^−4^ mol/L losartan), ML(high glucose plus 10^−5^ mol/L losartan), LL(high glucose plus 10^−6^ mol/L losartan), NL(normal glucose plus 10^−4^ mol/L losartan). Data are mean ± SEM (n = 4 for each group). *P<0.05, **P<0.01 compared with NG, #P<0.05, ##P<0.01 compared with HG.

**Figure 6:**
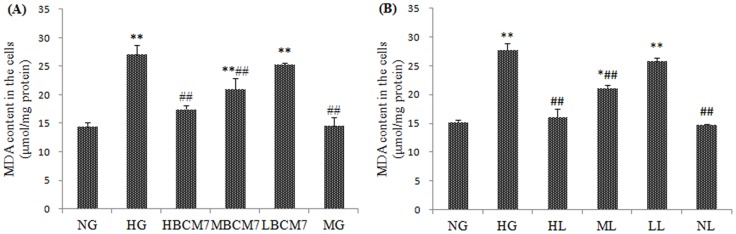
Content of MDA in the NRK-52E cells (n = 4). NG(normal glucose), HG(high glucose), HM(5.5 mmol/L glucose plus 24.5 mmol/L mannitol), HBCM7(high glucose plus 10^−5^ mol/L β-casomorphin-7),MBCM7(high glucose plus 10^−7^ mol/L β-casomorphin-7),LBCM7(high glucose plus 10^−9^ mol/L β-casomorphin-7), HL(high glucose plus 10^−4^ mol/L losartan), ML(high glucose plus 10^−5^ mol/L losartan), LL(high glucose plus 10^−6^ mol/L losartan), NL(normal glucose plus 10^−4^ mol/L losartan). Data are mean ± SEM (n = 4 for each group). *P<0.05, **P<0.01 compared with NG, #P<0.05, ##P<0.01 compared with HG.

As shown in Fig. 4A and Fig. 5A, the activity of SOD and GPx decreased significantly after exposure to HG for 72 h compared to that of NG. In contrast, the MDA level in HG treated cells increased significantly (Fig. 6A). Treatment of the cells with β-casomorphin-7 (10^−5^/10^−7^/10^−9^ mmol/L) obviously blunted the changes of SOD,GPx and MDA of NRK-52E cells in a concentration-dependent manner. Treatment of the cell with mannitol did not change the SOD GPx and MDA levels obviously compared to that of NG (Fig. 4A, 5A and 6A).

### Changes of Ang II in the kidney and culture medium

**Figure 7:**
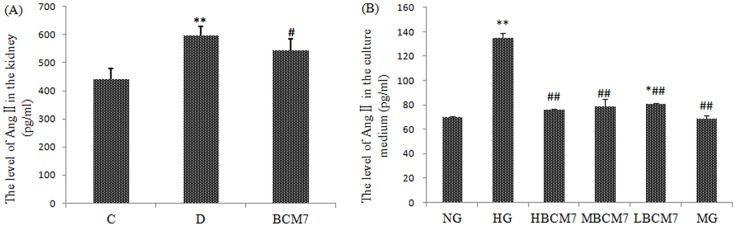
The level of Ang II in the kidney (n = 8) and NRK-52E cells (n = 4). Data are expressed as mean±SEM. (C) Control group; (D) Diabetic group; (BCM7) β-casomorphin-7 treatment group; NG(normal glucose), HG(high glucose), HM(5.5 mmol/L glucose plus 24.5 mmol/L mannitol), HBCM7(high glucose plus 10^−5^ mol/L β-casomorphin-7),MBCM7(high glucose plus 10^−7^ mol/L β-casomorphin-7),LBCM7(high glucose plus 10^−9^ mol/L β-casomorphin-7), **P<0.01, comparison between C group and D group. #P<0.05, comparison between D group and BCM7.

There was a significant increase of Ang II in the kidney of diabetic rats compared with the control group. BCM7 treatment reduced Ang II significantly in the kidney of diabetic rats (Fig. 7A).Moreover we found there was a significant increase of Ang II in the culture medium exposure to HG for 72 h compared to that of NG. β-casomorphin-7 treatment reduced Ang II significantly in the culture medium of NRK-52E cells exposure to HG. Treatment of the cell with mannitol (MG) failed to change Ang II level (Fig. 7B).

### Changes in the expression of ACE and ACE2 mRNA by real-time PCR

**Figure 8:**
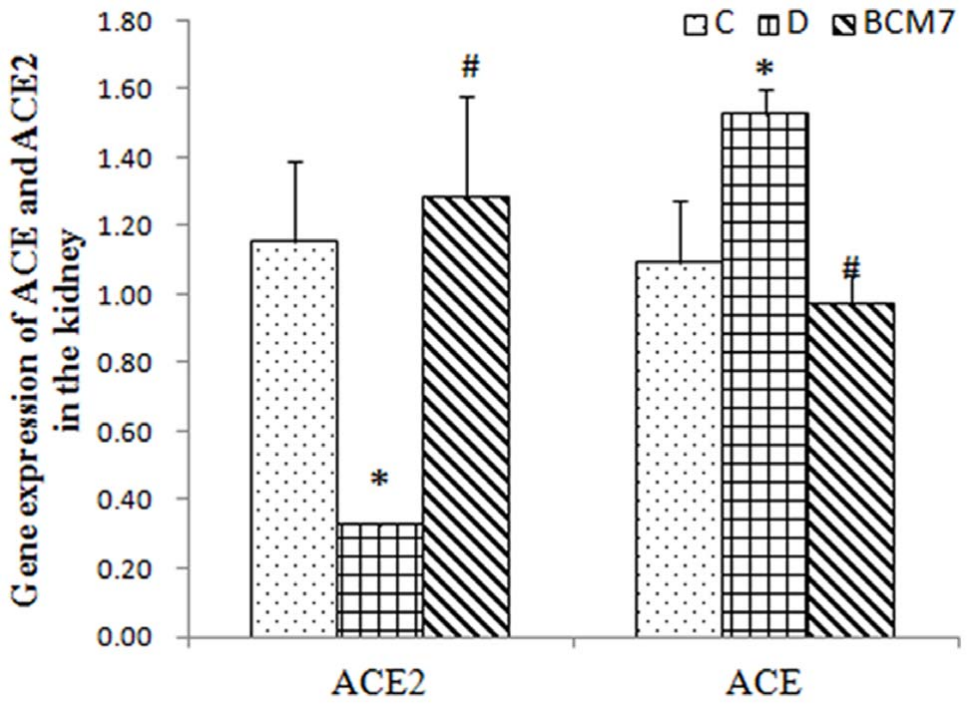
Changes of ACE2 and ACE in the kidney (n = 8). Data are expressed as mean±SEM (n = 8). (C) Control group; (D) Diabetic group; (BCM7) β-casomorphin-7 treatment group. *P<0.05, comparison between C group and D group. #P<0.05, comparison between D group and BCM7.

Real-time PCR revealed that compared to the rats of the control group, the diabetic rats had elevated expression of ACE mRNA and decreased expression of ACE2 mRNA. The expression of ACE mRNA was attenuated and ACE2 mRNA was increased by BCM7 treatment (Fig. 8).

### Changes of ACE and ACE2 expression by Western blot

**Figure 9:**
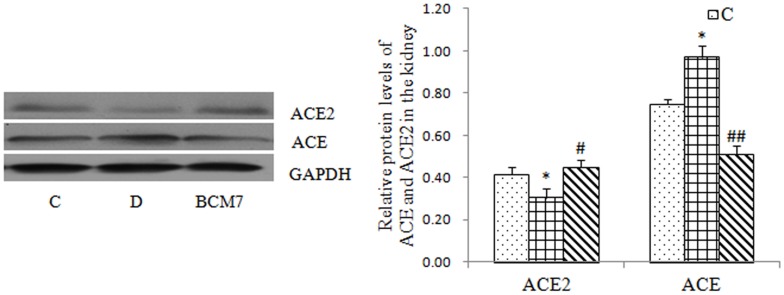
Changes of ACE and ACE2 protein in the kidney (n = 8). Data are expressed as mean±SEM (n = 8). (C) Control group; (D) Diabetic group; (BCM7) β-casomorphin-7 treatment group. *P<0.05, comparison between C group and D group. #P<0.05, comparison between D group and BCM7.

Compared to rats of the control group, diabetic rats had elevated ACE expression and decreased ACE2 expression in the kidney (Fig. 9).BCM7 treatment partially attenuated these effects, which is consistent with the expression of mRNA.

### Effects of losartan on HG-induced oxidative stress

Exposure of cells to HG for 72 h increased MDA levels compared with levels in cells exposed to NG (Fig. 6B), contrary, HG treatment decreased SOD and GPx activities (Fig. 4B and 5B). Losartan (10^−4^, 10^−5^, 10^−6^ mol/L) inhibited the HG-induced increase in MDA level and decrease in SOD and GPx activity in a concentration-dependent manner. Losartan(10^−4^ mol/L) failed to changed the SOD, GPx and MDA in the cells exposure to normal glucose(NL group, Fig. 4B, 5B and 6B).

## Discussion

Features of early diabetic renal injuries are glomerular hyperfiltration, glomerular and renal hypertrophy, increased urinary albumin excretion, increased basement membrane thickness and mesangial expansion[24]–[26]. It is in concert with our previous results that all diabetic rats in this experiment presented the early stage of DN, such as the increase of relative kidney weight, tubulointerstitial fibrosis, urinary albumin and urinary glucose after 30 days induction of STZ[13], while BCM7 treatment provided renoprotection with decreased urinary albumin and urinary glucose, alleviated tubulointerstitial fibrosis without changing blood glucose levels[13]. Our present study focused on evaluating the renoprotective effects of BCM7 to the oxidative stress in vivo and in vitro.

It has been reported in various studies that increased oxidative stress may play a role in the pathogenesis and progression of DN[4], [27]–[29].Both increased production of oxidants and decreased actions of antioxidants play roles in the increased oxidative stress in DN. MDA, a secondary product of lipid peroxidation, is a commonly used index of increased oxidative stress [20], [30], [31]. In our study we observed a significant increase in content of MDA in the kidney of diabetic rats and in the NRK-52E cells exposure to HG. This is consistent with the previous studies[6] and [7].The enzymatic activities(SOD and GPx), as the antioxidant indexes, decreased significantly in the kidney of diabetic rats and in the NRK-52E cells exposure to HG. These observations also are in agreement with the results of previous researches[7], [32]. Since an oxidative stress affects the cellular integrity only when antioxidant mechanisms are no longer able to cope with the free radical generation, supplementation of an antioxidant could gear up the detoxification machinery.

BCM7 was first isolated from an enzymatic digest of bovine casein[33]. Previous study found that BCM7 can protect rats from hyperglycemia and free radical-mediated oxidative stress in diabetic rats [12]. Moreover BCM7 can ameliorate deterioration of renal function and renal interstitial fibrosis caused by diabetes[13]. In our research we found BCM7 can increase the activity of SOD and GPx, and decrease the concentration of MDA in the kidney of diabetic rats and the NRK-52E cells exposure to HG (Table 2, Fig. 4A, 5A and 6A). All these data indicated that BCM7 can ameliorate high glucose-induced renal oxidative stress.

AngiotensinII (Ang II),which formed during hydrolyzing the angiotensin I (Ang I) by angiotensin converting enzyme (ACE) and can be hydrolyzed to produce Ang(1–7) by ACE2, is an important component of renin-angiotensin system (RAS). Ang II activation of the angiotensin type 1 receptor (AT1R) leads to deleterious effects such as oxidative stress and progressive renal dysfunction, that may ultimately lead to chronic kidney disease (CKD)[34]–[37]. In various models of experimental renal disease, an attenuation of oxidative stress following ACE inhibitors(ACEI) or AT1R antagonist (ARB) administration has been demonstrated[38]–[40]. Moreover ACE2 overexpression ameliorates diabetic renal oxidative stress[41]. All these amelioration of renal oxidative stress above is associated with the reduction of AngIIeffects. Our results showed that rats with STZ-induced diabetes had higher expression of ACE and lower expression of ACE2 in the kidney compared with the control rats. These changes have been ameliorated by BCM7 treatment. At the same time, our study demonstrated that BCM7 also reduced the level of AngII in the kidney of diabetic rats, which confirm the expression of ACE and ACE2.

Moreover we found that losartan (antagonist of angiotensinII type I receptor) could alleviate the decreases of SOD and GPx activity, the increase of MDA contents in the cells exposure to HG. We also found that BCM7 could attenuate the HG-induced increase in AngII in the culture medium in a concentration-dependent manner. It suggests that BCM7 may inhibit HG-induced oxidative stress by decreasing the level of AngII.

In summary, we demonstrated that of BCM7 effectively ameliorated HG-induced renal oxidative stress in a STZ-induced diabetic rat model and in the NRK-52E cells exposure to HG. The beneficial effects of BCM7 may contribute to reducing the contents of AngII in vivo and in vitro.
